# The impact of bullying victimization on non-suicidal self-injury in adolescents: the mediating role of rumination and the moderating role of friendship quality

**DOI:** 10.3389/fpsyg.2026.1627984

**Published:** 2026-01-30

**Authors:** Yanan Wang, Jing Wen, Qinghong Xu, Lu Zhang, Min Li

**Affiliations:** 1 School of Education, Inner Mongolia Minzu University, Tongliao, China; 2 Inner Mongolia Student Bullying Prevention Research Center, Tongliao, China; 3 Inner Mongolia Ethnic Education and Psychological Development Research Base, Tongliao, China; 4 School of Foreign Languages, Yulin University, Yulin, China

**Keywords:** adolescent, bullying victimization, friendship quality, non-suicidal self-injury, rumination

## Abstract

Non-suicidal self-injury (NSSI) is a prevalent global public health concern among adolescents, with bullying victimization recognized as a key risk factor, while the underlying cognitive mechanisms and interpersonal protective factors remain understudied. This study aimed to investigate (1) the relationship between bullying victimization and non-suicidal self-injury (NSSI) in adolescents, (2) the mediating role of rumination in the association between bullying victimization and NSSI, and (3) the moderating role of friendship quality in the relationship between rumination and NSSI. A sample of 692 adolescents was assessed using the Bullying Victimization Questionnaire, the Adolescent NSSI Behavior Assessment Questionnaire, the Ruminative Responses Scale (RRS), and the Friendship Quality Questionnaire (FQQ). Results indicated that: (1) Bullying victimization exerted a significant positive predictive effect on NSSI (explaining 59.85% of the variance); (2) Rumination partially mediated the link between bullying victimization and NSSI, accounting for 40.15% of the total effect; (3) Friendship quality moderated the relationship between rumination and NSSI (*β* = −0.002, *p* < 0.001), attenuating the detrimental impact of rumination on NSSI. These findings collectively suggest that bullying victimization, rumination, and lower friendship quality collectively heighten adolescents’ risk of engaging in NSSI.

## 1 Introduction

### 1.1 Adolescent NSSI: behaviors, impacts and intervention highlights

Non-Suicidal Self-Injury (NSSI) refers to the deliberate, self-inflicted damage to bodily tissue without suicidal intent (Nock, 2009). Common manifestations include cutting, burning, hitting, and other forms of self-harm, often employed by individuals to alleviate negative emotions, seek social attention, or express internal distress (Hooley et al., 2020). NSSI is particularly prevalent among adolescents and constitutes a global public health concern. International studies indicate that the lifetime prevalence of NSSI in adolescents is approximately 17.2% (Swannell et al., 2014), while in China, reported rates range from 7.5 to 46.5% (Zhou and Jiang, 2021). Beyond physical harm, NSSI exacerbates psychological burdens, potentially triggering or intensifying anxiety, depression, and related mental health disorders (Gratz, 2001). During adolescence, individuals face intense academic competition, heightened sensitivity to peer approval, and underdeveloped cognitive control, rendering them more vulnerable to adopting maladaptive coping strategies like NSSI when confronted with adversity (Espelage et al., 2003). Notably, NSSI frequency tends to decline with age, underscoring adolescence as a critical window for intervention (Daukantaitė et al., 2021). Given its detrimental consequences, widespread prevalence, and time-sensitive intervention potential, NSSI has garnered considerable research attention in adolescent mental health studies.

### 1.2 The related of bullying and non-suicidal self-injury

Prior research has established bullying victimization as a significant risk factor for non-suicidal self-injury (NSSI). School bullying, defined as repeated and intentional physical or psychological aggression by one or more students toward a peer, resulting in physical or emotional harm (Olweus, 1994), disproportionately increases NSSI susceptibility among victims. Adolescents with bullying experiences exhibit significantly higher rates of NSSI compared to non-victimized peers, a pattern amplified by developmental vulnerabilities such as psychological fragility and immature self-regulation capacities during adolescence (Wen et al., 2023). Bullying victimization may trigger maladaptive responses across cognitive, emotional, and behavioral domains, heightening the likelihood of adopting NSSI as a coping mechanism. The General Strain Theory (Agnew, 1992) provides a robust framework for understanding this relationship. GST posits that exposure to chronic stressors (e.g., bullying) generates oppressive strain when individuals lack adaptive coping resources. To alleviate such strain, adolescents may resort to deviant or self-destructive behaviors, including NSSI. Bullying, as a persistent school-based stressor, exerts cumulative negative effects on victims, fostering emotional dysregulation and normalizing self-harm as an escape strategy.

### 1.3 The mediating role of rumination

Following exposure to bullying, victims frequently experience intense negative emotions. When such emotions persist unresolved, they may trigger recurrent intrusive thoughts about the bullying episode, fostering the development of ruminative processes (Labella et al., 2024). Rumination perpetuates immersion in dysphoric affective states, which—when prolonged and unregulated—overwhelm individuals’ coping capacities, driving them to seek emotional relief through maladaptive behaviors such as non-suicidal self-injury (NSSI) (Selby et al., 2013). The Emotional Cascade Model (Selby et al., 2009) provides a mechanistic framework for understanding the rumination-NSSI linkage. This model posits that rumination amplifies emotional reactivity through cyclical cognitive-affective feedback loops, trapping individuals in escalating distress. To disrupt this cascade, individuals may engage in NSSI, as the physical pain generated by self-injury temporarily diverts attention from ruminative content, thereby attenuating emotional intensity. Over time, NSSI becomes a reinforced behavioral pattern due to its perceived efficacy in terminating rumination. Among bullied adolescents, the inability to process trauma-related emotions adaptively increases vulnerability to NSSI as a misguided coping strategy. By misattributing transient relief from self-harm as functional emotion regulation, victims may establish a conditioned response wherein future stressors activate NSSI automatisms (Nock, 2010). This maladaptive cycle underscores the critical need for interventions targeting rumination reduction and alternative coping skill development in bullied youth.

### 1.4 The moderating role of friendship quality

Friendship, as a major developmental task in adolescence (Poulin and Chan, 2010), plays an important role in subjective well-being (Bagwell et al., 2015). Friendships typically exhibit characteristics of permanence, intimacy, and stability (Bukowski and Hoza, 1989). Friendship Quality (FQ) describes the state of a friendship relationship, referring to both the inherent characteristics of the friendship (e.g., emotional support, conflict resolution) and a key indicator of its adaptive value for adolescents (Parker and Asher, 1993)—a definition that has been refined and extended by recent research. In digitalized and socially complex adolescent contexts, contemporary studies (e.g., Eggermont et al., 2021) have supplemented this conceptualization by highlighting that high-quality friendships now also include consistent peer responsiveness (even in online interactions) and mutual validation of distress—dimensions that align with the “affirmation and concern” and “intimate disclosure and communication” dimensions of the Friendship Quality Questionnaire (FQQ) used in this study. These updated perspectives reinforce that FQ remains a multi-faceted construct centered on emotional and interpersonal support, which is consistent with our measurement framework.

During adolescence, individuals become increasingly emotionally dependent on the support of close friends (Furman and Buhrmester, 1992). High friendship quality not only provides adolescents with external social support (e.g., peer care), but also helps to enhance their internal self-worth (e.g., self-identity), which enables adolescents to cope with setbacks and failures positively, thus facilitating their growth and development (Troop-Gordon et al., 2019).

It has been shown that positive peer association promotes growth in positive affect and negative peer association promotes growth in negative affect (Jose, 2015). High-quality friendships can provide individuals with emotional support and help them break out of the rumination cycle (Rose et al., 2007). That is, when an individual is caught up in repeated thoughts about a negative event, the empathic listening of a friend can help him or her feel understood and supported, thus relieving emotional stress.

High-quality friendships may reduce the risk of NSSI behaviors by reducing rumination (Wang et al., 2020). Low-quality friendships may act as a source of interpersonal stress, leading individuals to develop non-suicidal self-injurious behaviors (Giletta et al., 2015). It has been shown that there is a significant association between friendship quality and non-suicidal self-injury, and that adolescents with lower intimacy, helpfulness, and friendship security may be more prone to non-suicidal self-injurious behaviors (Eggermont et al., 2021). In contrast, high-quality friendships may reduce the risk of NSSI by providing emotional support and facilitating effective emotion regulation strategies that help individuals reduce rumination thinking.

Despite the clear connections between bullying victimization, rumination, friendship quality, and NSSI in existing research, several key limitations remain unaddressed. First, most studies have focused either on direct links between individual factors and NSSI or on single mediating or moderating effects, rather than combining cognitive processes (like rumination) and interpersonal protective factors (like friendship quality) into a single, cohesive framework. Second, research on friendship quality has mostly viewed it as a general safeguard against NSSI, without exploring how it might specifically ease the process through which bullying leads to NSSI via rumination. Third, few studies have examined whether the role of rumination changes depending on friendship quality—a detail that is crucial for designing targeted interventions. These gaps make it hard to fully understand the multi-layered mechanisms behind NSSI in bullied adolescents, and they limit the ability to create effective prevention strategies.

**Figure 1:**
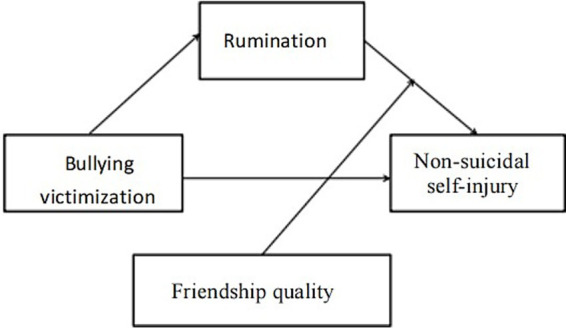
Conceptual model.

Based on the comprehensive analysis of theoretical and empirical research, this study explored the relationship between adolescents’ bullying experience, rumination thinking, friendship quality and non-suicidal self-injury (NSSI) from multiple perspectives. Based on the above research, it is hypothesized that bullying experience is not only directly related to non-suicidal self-injurious behavior, but may also indirectly affect the occurrence of NSSI through rumination thinking. Meanwhile, friendship quality plays a moderating role in the relationship between rumination thinking and NSSI, which may alleviate the negative emotions triggered by rumination thinking and thus reduce the probability of NSSI. Accordingly, this study proposes a path-relationship model containing bullying experience, rumination thinking, friendship quality, and NSSI (see Figure 1). The model aims to elucidate the psychological process by which bullying experience leads to NSSI via rumination thinking, and to examine the moderating role of friendship quality in this process, providing a theoretical basis for adolescent mental health intervention and bullying prevention.

## 2 Research purpose

The purpose of this study was to construct a mediated moderation model (see Figure 1) to systematically investigate the relationship between bullying experiences and adolescent nonsuicidal self-injury. We tested the following hypotheses: H1: Bullying experiences have a direct and significant effect on nonsuicidal self-injurious behaviors. H2: Rumination thinking mediates the relationship between bullying experiences and adolescent nonsuicidal self-injury and exacerbates the risk of nonsuicidal self-injury. H3: Friendship quality moderates the relationship between rumination thinking and adolescent nonsuicidal self-injury and mitigates the risk of nonsuicidal self-injury. Examining the mediating and moderating mechanisms of the relationship between bullying experiences and NSSI helps to provide a solid theoretical foundation for future intervention programs.

This study’s design and hypotheses are rooted in classic, textbook-referenced theories in adolescent psychology and mental health, which collectively underpin the logical pathways of our model. General Strain Theory—core to understanding stress-behavior links—posits that chronic stressors like bullying victimization trigger deviant behaviors (e.g., NSSI) when adaptive coping is lacking, justifying our hypothesis that bullying directly predicts NSSI (H1). The Emotional Cascade Model, a foundational framework for cognition-emotion interactions, explains how rumination amplifies distress and drives NSSI, supporting our mediating hypothesis (H2). Additionally, Ecological Systems Theory, which emphasizes individual-environment interactions, rationalizes including friendship quality as a moderator (H3)—as positive interpersonal contexts (e.g., high-quality friendships) can buffer rumination’s adverse effects. These established theories form the cornerstone of our “bullying victimization → rumination → NSSI” model with friendship quality moderation, ensuring rigorous theoretical alignment.

## 3 Research methodology

### 3.1 Inclusion/exclusion criteria

Inclusion criteria: (1) Enrolled in the first to third grade of junior high school; (2) Ability to understand the questionnaire correctly and cooperate with the survey; (3) Consent of the subjects and their families.

Exclusion criteria: (1) Presence of neurological or other psychiatric diseases; (2) Presence of drug or alcohol dependence; (3) People suffering from epilepsy, traumatic brain injury, or mental deficiencies; (4) People who have suffered from a major stressful event in the recent past;(We clarified the concept of major stressful events to students as events causing sustained emotional distress for ≥ 2 weeks, and asked students to anonymously check the relevant option at the end of the questionnaire.) (5) People who suffer from hearing and speech disorders, etc., which affect normal communication; (6) People who are unable to cooperate with the survey.

### 3.2 Participants

Adolescent students from six middle schools in Tongliao City, Inner Mongolia, participated in the questionnaire survey. A total of 826 questionnaires were distributed. The survey was conducted in a face-to-face, self-administered paper-and-pencil format: researchers entered the classrooms, explained the purpose and confidentiality of the survey to the students, and then distributed the paper questionnaires, which the students completed independently on the spot. After careful screening, 125 questionnaires were excluded: A total of 826 questionnaires were distributed in this study. Finally, 125 samples were excluded, all due to missing key items in the core scales, which did not meet the requirements for data integrity of the study. A total of 692 valid samples were obtained, with a validity rate of 84.7%. This resulted in 692 valid participants and a validity rate of 84.7%. The information of valid participants is shown in Table 1.

**Table 1:** Statistics on the number of boys and girls in each grade.

Grade	Male	Female	Total	Percentage
First grade	27	36	63	9.0%
Second grade	69	49	118	16.8%
Third grade	72	72	144	20.5%
Freshman year	80	70	150	21.4%
Sophomore year	66	77	143	20.4%
Senior year	24	50	74	10.6%
Total	338	354	692	100%

The period of data collection and entry in this study was from November 2023 to January 2024.

### 3.3 Research tools

#### 3.3.1 Bullying questionnaire

This study adopted the bullying scale from the Olweus Bullying Questionnaire revised by Zhang and Wu (1999). The scale consists of 6 questions and contains three dimensions: verbal bullying, physical bullying, and relational bullying. The scale is scored on a 5-point Likert scale, with 0 representing “never” and 4 representing “almost every day” (note: a 5-point scale typically ranges from 0 to 4, aligning with 5 response options), and the average score is calculated. Higher scores indicate higher levels of bullying victimization. The questionnaire has good reliability and validity, with a Cronbach’s alpha coefficient of 0.79 for this scale in the current study.

#### 3.3.2 Non-suicidal self-injury questionnaire

Developed by Wan et al. (2018) to obtain information on the occurrence of 12 self-injurious behaviors in the study population in the last 1 year. The total questionnaire was divided into a functional questionnaire and a behavioral questionnaire. The questionnaire consists of two parts, the behavioral questionnaire and the functional questionnaire, of which the behavioral questionnaire consists of 12 questions and the functional questionnaire consists of 19 questions. The scale consists of two dimensions: no obvious tissue damage and obvious tissue damage, with a 5-point scale, where 0 stands for “never happened” and 5 stands for “almost every day,” and the total number of points is calculated, and the higher the score, the more serious the non-suicidal self-injury behavior is. The questionnaire has good reliability and validity, and the Cronbach’s alpha coefficient of the NSSI Behavior Rating Questionnaire for Adolescents is 0.74.

#### 3.3.3 Ruminate thinking scale (RRS)

The Rumination Thinking Scale developed by Nolen-Hoeksema et al. (2008) and Morrow and revised by Han and Yang (2009) was used. The revised scale consists of 22 items, divided into three dimensions: symptomatic rumination dimension, obsessive thinking dimension, and introspective thinking dimension, with a 4-point scale and a total number of scores calculated, with higher scores representing a more serious tendency to ruminate. The questionnaire has good reliability and validity, and the Cronbach’s alpha coefficient of the Rumination Thinking Scale (RRS) is 0.90.

#### 3.3.4 Friendship quality scale(FQQ)

The Friendship Quality Questionnaire revised by Han and Yang (2009) was used. The questionnaire consists of 18 questions, including six dimensions: affirmation and concern, help and guidance, intimate disclosure and communication, companionship and recreation, conflict resolution strategies, and conflict and betrayal, and is scored on a 5-point scale ranging from “1” for “not at all consistent” to “5” for “completely consistent.” A 5-point scale was used, from “1” for “not at all consistent” to “5” for “completely consistent” (consistent with the above scoring criteria), and the total scores on all questions were calculated, with higher scores indicating better quality of the individual’s friendships. The reliability of the questionnaire was good, and the Cronbach’s alpha coefficient of the Friendship Quality Scale (FQQ) was 0.88. The FQQ is a measure of the quality of friendship between individuals.

### 3.4 Procedures

This study was approved by the Research Ethics Committee of Inner Mongolia University for Nationalities. The researcher introduced the purpose and content of the study after establishing contact with teachers from two middle schools in Naiman Banner, Tongliao City. After obtaining consent, the local school teachers used a paper version of the questionnaire and administered it to the whole class after school hours—during which, to ensure the authenticity of responses to sensitive topics (e.g., bullying victimization, NSSI), classroom teachers were requested to leave the classroom once the survey began, and only the trained researchers (who had received unified training on standardized instruction language and response guidance, and were unrelated to the participating schools) remained on-site. These researchers only answered procedural questions (e.g., “How to interpret scale items”) and did not interfere with participants’ independent responses, further ensuring the standardization of the data collection process.

The students completed the questionnaire anonymously, and written informed consent and assent were obtained from the participants and their legal guardians/next of kin prior to the administration of the questionnaire. They participated voluntarily and adhered to the assurance of confidentiality; to further protect response privacy, completed questionnaires were collected in sealed envelopes to avoid mutual viewing between participants. Completion of the self-report questionnaire took approximately 30 min.

### 3.5 Data analysis

In this study, common method bias test, descriptive statistical analysis, and correlation analysis were performed on the collected data using SPSS 26.0; mediation model analysis and moderated mediation model analysis were performed through Model 4 and Model 14, respectively, in the SPSS Process macro program developed by Hayes. Gender and grade level were included as covariates in this study.

## 4 Results

### 4.1 Descriptive statistics and correlation analysis

Table 2 presents the mean and standard deviation of the variables and their correlation coefficients. After analysis, it was found that there was a significant correlation among bullying experience, rumination, friendship quality, and non-suicidal self-injury, with friendship quality being negatively correlated with non-suicidal self-injury, bullying experience, and rumination. This suggests that the higher the level of friendship quality, the lower the likelihood of non-suicidal self-injury occurring; individuals with a high level of friendship quality had a low score for rumination; and the lower the score for friendship quality, the more likely adolescents were to experience bullying.

**Table 2:** Descriptive statistics and correlations between variables.

Variable	*M* ± *SD*	1	2	3	4	5	6
1. Gender	1.510 ± 0.500	–					
2. Grade	3.600 ± 1.478	0.081*	–				
3. Bullying victimization	1.135 ± 0.305	−0.123**	−0.029	–			
4. Rumination	42.449 ± 12.008	0.085*	0.144***	0.201***	–		
5. Friendship quality	64.009 ± 12.300	0.103**	0.054	−0.198***	−0.154***	–	
6. Non-suicidal self-injury	13.562 ± 3.779	−0.026	−0.083*	0.215***	0.394***	−0.124**	–

*n* = 692; *M*, mean; *SD*, standard deviation; *p < 0.05, **p < 0.01, ***p < 0.001.

Other than that, all other pairs showed significant positive correlations, which indicated that the more frequent the bullying, the more severe the adolescents’ rumination; the more severe the bullying, the more severe the adolescents’ non-suicidal self-injurious behaviors; and the higher the level of rumination, the higher the likelihood of non-suicidal self-injurious behaviors. The relationships between variables supported further testing of subsequent hypotheses. In addition, we found a significant negative correlation between grade and NSSI (r = −0.083, *p* < 0.05), which is in line with the core developmental characteristics of adolescents. As grade increases, adolescents’ emotional regulation abilities gradually mature—they are more likely to adopt adaptive coping strategies (such as seeking support or cognitive adjustment) when facing bullying-related distress, instead of relying on impulsive NSSI. Meanwhile, higher-grade adolescents often have more stable and in-depth social networks, and the protective effect of friendship quality is more prominent, which further reduces the risk of NSSI.

### 4.2 Test of the mediating model of rumination

**Figure 2:**
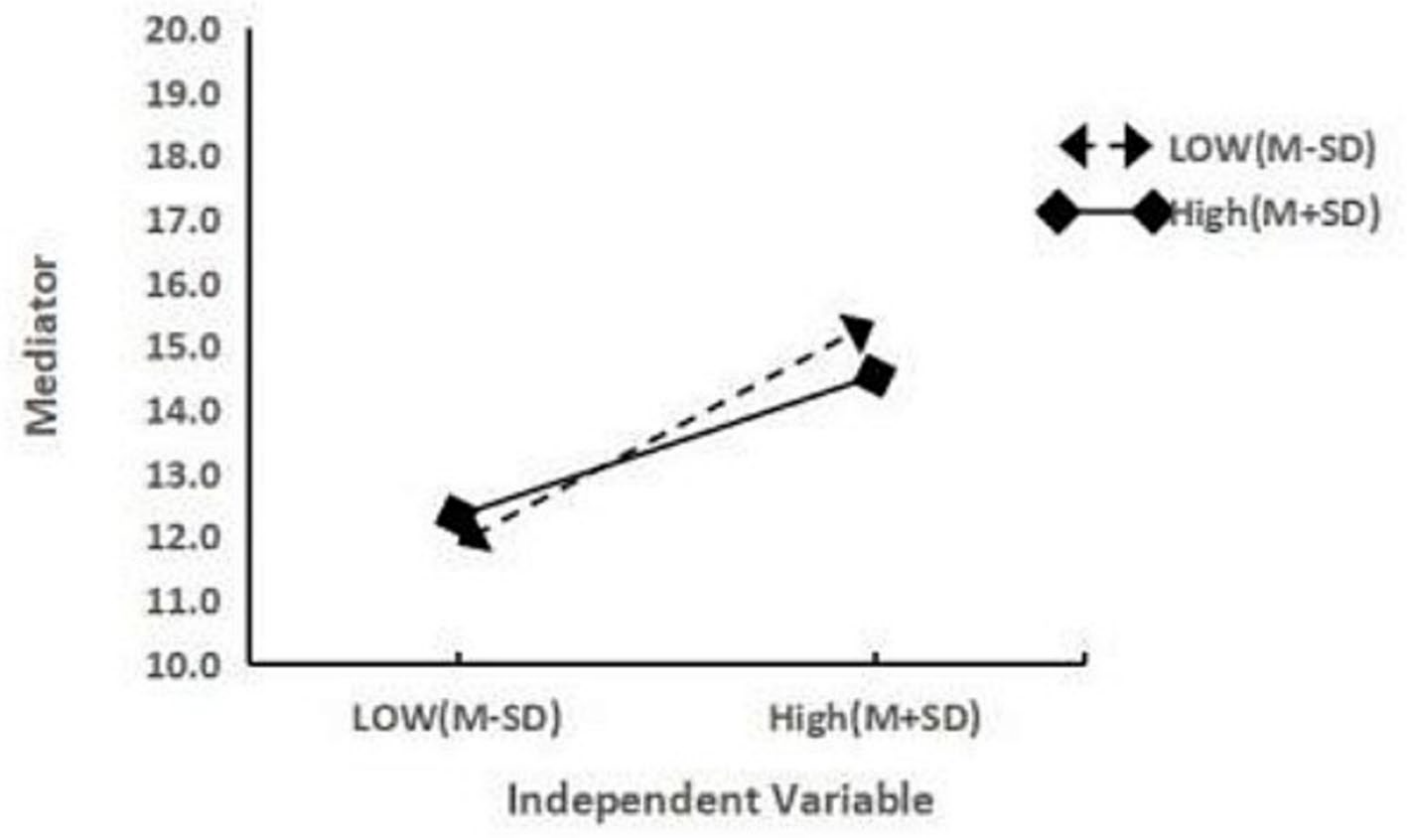
Friendship quality moderating the relationship between rumination and non-suicidal self-injury.

The presence of a mediation model was tested using Model 4 of Process in SPSS. After controlling for the effects of gender and grade, bullying experience was positively correlated with non-suicidal self-injury (*β* = 8.543, *p* < 0.001). As shown in Figure 2, bullying experience was positively correlated with non-suicidal self-injury when rumination was included (*β* = 1.577, *p* < 0.001), and rumination was positively correlated with non-suicidal self-injury (*β* = 0.124, *p* < 0.001) (see Table 3).

**Table 3:** Mediation model of rumination between bullying victimization and non-suicidal self-injury.

Predictors	Rumination			Non-suicidal self-injury		
	*β*	SE	95%*CI*	*β*	SE	95%*CI*
Constant	25.015***	2.490	[20.126, 29.904]	8.115***	0.791	[6.562, 9.668]
Gender	2.442**	0.887	[0.700, 4.183]	−0.257	0.264	[−0.777, 0.262]
Grade	1.152***	0.298	[0.567, 1.74]	−0.342**	0.089	[−0.518, −0.167]
Bullying victimization	8.543***	1.441	[5.713, 11.373]	1.577***	0.438	[0.717, 2.438]
Rumination				0.124***	0.011	[0.102, 0.147]
*R* ^2^		0.074			0.194	
*F*		18.259***			41.381***	

*n* = 692; Bootstrap sample size, 5,000; CI, confidence interval; *β*, standardized coefficient; *p < 0.05; **p < 0.01; ***p < 0.001.

The Bootstrap test found a mediating effect value of 1.063, with a 95% confidence interval of [0.628, 1.670]; the interval did not include zero, and the indirect effect accounted for 40.15% of the total effect. Thus, ruminative thinking partially mediates the relationship between bullying experiences and non-suicidal self-injury, and Hypothesis 2 was supported (Table 4).

**Table 4:** Bootstrapping indirect effect and 95% confidence interval (CI) for the mediation model: victimization and non-suicidal self-injury through rumination.

	Estimated effect	*SE*	95%*CI*	Ratio to total effect
Total effect	2.640	0.463	[1.731, 3.550]	
Direct effect	1.557	0.438	[0.717, 2.438]	59.85%
Indirect effect	1.063	0.266	[0.628, 1.670]	40.15%

*n* = 692; Bootstrap sample size, 5,000; CI, confidence interval.

### 4.3 Test of the moderating model of friendship quality

The mediated moderation model was tested using Model 14 of Process in SPSS. From Table 5, it can be seen that the interaction term of rumination thinking and friendship quality significantly influenced non-suicidal self-injury (*β* = −0.002, *p* < 0.001).

**Table 5:** Moderated mediation model of friendship quality between bullying victimization and non-suicidal self-injury through rumination.

Predictors	Rumination			Non-suicidal self-injury		
	*β*	*SE*	95%*CI*	*β*	*SE*	95%*CI*
Constant	25.015***	2.490	[20.126, 29.904]	2.862	2.349	[−1.749, 7.474]
Gender	2.442**	0.887	[0.700, 4.183]	−0.198	0.264	[−0.717, 0.321]
Grade	1.152***	0.298	[0.567, 1.737]	−0.342***	0.089	[−0.517, −0.169]
Bullying victimization	8.543***	1.441	[5.713, 11.373]	1.326***	0.446	[0.450, 2.202]
Rumination				0.264***	0.050	[0.166, 0.362]
Friendship quality				0.087**	0.035	[0.018, 0.156]
Rumination × Friendship quality				−0.002***	0.001	[−0.004, −0.001]
*R* ^2^		0.074			0.205	
*F*		18.259***			29.446***	

*n* = 692; Bootstrap sample size, 5,000. CI, confidence interval; *β*, standardized coefficient; *p < 0.05; **p < 0.01; ***p < 0.001.

To further explain the moderating effect of friendship quality, friendship quality was divided into high and low groups according to its mean plus or minus one standard deviation (M ± SD), and a simple slope test was performed (see Figure 2). The results showed that ruminative thinking was a significant predictor of non-suicidal self-injury when friendship quality scores were low (Effect = 1.258, 95% CI [0.712, 1.966]), and a significant but significantly weaker predictor of non-suicidal self-injury when friendship quality scores were high (Effect = 0.789, 95% CI [0.416, 1.343]). In other words, the effect of rumination on non-suicidal self-injury diminished as the friendship quality score increased. Hypothesis 3 was supported. Additionally, the inclusion of the rumination × friendship quality interaction term increased the model’s *R*^2^ by 0.010 (*R*^2^-chng = 0.010, *F* = 8.486, *p* = 0.004), with the overall *R*^2^ of the moderated model being 0.205. The semi-partial f^2^ for the interaction was calculated as 0.010/(1–0.205) ≈ 0.013, indicating a small but statistically significant effect of the interaction.

Further Johnson–Neyman analysis revealed that no statistical significance transition points existed within the observed range of friendship quality in this study. This indicates that the positive predictive effect of rumination on NSSI remained significant across all levels of friendship quality, but its effect size gradually weakened as friendship quality increased—consistent with the simple slope results.

## 5 Discussion

### 5.1 Bullying and non-suicidal self-injury

The present study found that adolescent bullying significantly and positively predicted non-suicidal self-injury, which is consistent with previous findings and further confirms the impact of bullying on non-suicidal self-injury (Wen et al., 2023). This may be due to the fact that many individuals who have experienced bullying have difficulties in emotional regulation (Wu, 2021); they struggle to cope with negative emotions adaptively (Vacca et al., 2023) and thus choose self-injurious behaviors as a coping mechanism to release inner pain and tension, thereby gaining a sense of comfort and relaxation (Van Geel et al., 2015). As a type of childhood trauma, adolescent bullying may lead to the emergence of psychological problems such as depression (Rengasamy et al., 2021). These psychological problems cause individuals to develop negative perceptions and emotions toward themselves, prompting them to alleviate such psychological distress through self-injury.

### 5.2 The mediating role of rumination in bullying and non-suicidal relationships

The present study found that ruminative thinking partially mediates the relationship between bullying and adolescent non-suicidal self-injury, suggesting that bullying behavior not only directly predicts non-suicidal self-injury, but also indirectly predicts non-suicidal self-injury through the mediating role of ruminative thinking. When individuals are bullied, those with high levels of ruminative thinking constantly replay scenes and details of the bullying in their minds, and this repeated recollection reinforces their negative perceptions of their experiences (Malamut and Salmivalli, 2021). According to the Emotional Cascade Model, intense rumination leads to the accumulation of negative emotions, which may trigger self-injurious behaviors (Selby et al., 2010); self-injurious behaviors can serve to inhibit the emotional cascade by providing strong physical sensations to interrupt rumination (Selby et al., 2010). In other words, rumination can lead to prolonged psychological distress in bullied individuals, and when this distress reaches a certain level and cannot be relieved by other means, they may resort to non-suicidal self-injurious behaviors as a coping strategy.

### 5.3 Moderating effect of friendship quality

The results of the present study indicate that friendship quality moderates the effect of rumination on non-suicidal self-injury, as evidenced by the fact that the higher the level of friendship quality, the weaker the positive predictive effect of rumination on non-suicidal self-injury. This is generally consistent with the results of previous studies. High-quality peer relationships help individuals develop sound emotional regulation (Reavis et al., 2015). Emotional support provided by high-quality friendships can activate individuals’ social comparison and emotion-sharing mechanisms, prompting ruminative thinking to shift from internal circulation to external expression. Specifically, the peer communication process not only reduces the level of emotional arousal through the buffering effect of social support (Cohen and Wills, 1985), but also reconfigures the interpretation of the meaning of negative events through cognitive reappraisal strategies—through interactive discussions with friends, individuals are able to reappraise the threat of stressors from multiple perspectives.

Combined with the Emotional Cascade Model used in this study, two possible action paths of friendship quality are proposed: First, high-quality friendships can reduce the frequency of rumination—when adolescents share bullying-related distress with supportive friends, timely emotional validation and listening can interrupt the cycle of intrusive and repetitive negative thoughts, preventing rumination from escalating. Second, high-quality friendships may change the content of rumination: supportive friends can help adolescents reframe negative experiences, shifting self-blaming thoughts (e.g., “I deserve to be bullied”) to more adaptive appraisals (e.g., “This is not my fault and I have support”), thereby reducing the emotional intensity of rumination and weakening its predictive effect on NSSI. Future longitudinal studies can further verify these two paths to clarify the specific mechanism of friendship quality’s protective effect.

Through interactive discussions with friends, individuals are able to reevaluate the threat level of a stressor from multiple perspectives, and this cognitive reframing process significantly reduces the intrusiveness and persistence of rumination (Nolen-Hoeksema et al., 2008). When the intrusiveness and persistence of ruminative thinking are reduced, the individual’s psychological distress caused by rumination is correspondingly alleviated, which in turn reduces the intrinsic drivers that trigger non-suicidal self-injury, thereby decreasing the occurrence of non-suicidal self-injury.

### 5.4 Implications for theory, research and practice

This study explored the relationship between bullying and non-suicidal self-injury (NSSI), revealing the mediating role of rumination and the moderating role of friendship quality. Findings suggest that bullying increases adolescents’ non-suicidal self-injurious behaviors through ruminative thinking, and that high-quality friendships buffer this effect. This finding provides an important theoretical basis and practical guidance for preventing and intervening in adolescents’ non-suicidal self-injurious behaviors. Meanwhile, the results of this study support a variety of theoretical models, such as the General Stress Theory, the Emotion Management Model, and the Interpersonal Relationship Model. These models provide a multidimensional perspective for understanding adolescent non-suicidal self-injurious behavior, emphasizing the interaction between environmental and individual factors.

The results of the study showed that ruminative thinking played an important mediating role between bullying experiences and adolescent non-suicidal self-injury. This result is consistent with the Emotional Cascade Model, in which ruminative thinking interacts with negative emotions to form a vicious cycle that ultimately leads to non-suicidal self-injurious behavior. Therefore, understanding and intervening in ruminative thinking is crucial for preventing non-suicidal self-injurious behavior in adolescents. In this regard, schools should strengthen mental health education to help adolescents identify and manage their emotions, especially ruminative thinking. Emotion regulation strategies and coping skills can be provided through mental health classes, workshops, and counseling activities. For adolescents with ruminative thinking and non-suicidal self-injurious behaviors, interventions can be implemented using Cognitive Behavioral Therapy (CBT), which can help adolescents identify and change negative thinking patterns and learn more effective coping strategies.

Friendship quality plays a moderating role between ruminative thinking and non-suicidal self-injury. High-quality friendships can provide emotional support and help adolescents better cope with the negative emotions associated with bullying, thereby reducing the occurrence of non-suicidal self-injurious behaviors. Schools should strive to create a positive, supportive environment that reduces the incidence of bullying. They can enhance students’ sense of belonging and safety through anti-bullying activities, the establishment of peer support groups, and the provision of counseling services. Additionally, schools can help youth establish and maintain high-quality friendships and strengthen their social support networks through social skills training.

To further clarify the practical value of these findings—beyond the established understanding that bullying and rumination are key intervention targets—three actionable insights emerge. First, regarding the moderating effect of friendship quality, our simple slope analysis quantifies its real-world impact: when friendship quality was 1 standard deviation below the mean (low quality), rumination’s predictive effect on NSSI was 1.258 (95% CI [0.712, 1.966]); when friendship quality was 1 standard deviation above the mean (high quality), this effect weakened by approximately 37% (effect = 0.789, 95% CI [0.416, 1.343]). This means even modest improvements in friendship quality (e.g., helping adolescents build one stable, supportive peer relationship) can meaningfully reduce NSSI risk for those with high rumination.

Second, we provide operational thresholds for screening high-risk groups: adolescents with bullying victimization scores above the sample mean (M = 1.135, SD = 0.305), rumination scores above the mean (M = 42.449, SD = 12.008), and friendship quality scores below the mean (M = 64.009, SD = 12.300) can be prioritized for early intervention—addressing the lack of targeted screening tools in current practice.

Third, these findings optimize existing programs: schools can supplement anti-bullying efforts with brief rumination awareness activities (e.g., teaching students to redirect repetitive negative thoughts) and friendship-building exercises (targeting intimacy or conflict resolution); clinicians can integrate social skill training (to boost friendship quality) into CBT for rumination, rather than focusing solely on cognitive restructuring.

Regarding the small interaction term coefficient (B ≈ −0.002), its significance lies in cumulative, contextual effects: the interaction’s effect size (*f*^2^ = 0.026) is “small but meaningful” for social science (Cohen and Wills, 1985), and its stability (*p* < 0.001) confirms it is not random. Over time, even small reductions in rumination—driven by supportive friendships—prevent emotional escalation to NSSI. Additionally, friendship quality acts as a low-resource complement to other interventions (e.g., anti-bullying policies), forming a sustainable multi-layered protection system.

In terms of intervention scenarios, this model is applicable to both primary prevention (targeting all adolescents—e.g., school-wide friendship-building programs and rumination awareness curricula to reduce overall NSSI risk) and secondary prevention (targeting bullied adolescents with high rumination and low friendship quality—e.g., targeted social skill training and cognitive restructuring to interrupt the “bullying → rumination → NSSI” pathway).

### 5.5 Practical implications in clinical and community settings and advantages over previous research

Compared with prior studies that often focused on single links between bullying and NSSI or isolated mechanisms, this research offers key advantages: it constructs a holistic moderated mediation model, clarifying how rumination (a cognitive factor) and friendship quality (an interpersonal factor) interact to shape NSSI risk—filling gaps in multi-level mechanism exploration. Additionally, the large, gender-balanced sample (692 adolescents across middle and high schools) enhances result generalizability, addressing limitations of smaller, homogeneous samples in earlier work.

In practice, these findings solve core challenges in adolescent mental health: clinically, they guide targeted interventions (e.g., using cognitive restructuring in CBT to reduce rumination, while helping bullied youth build high-quality friendships as emotional buffers); in communities, they support low-resource strategies like peer support groups (leveraging friendship quality) and rumination management workshops (e.g., mindfulness training), which are accessible even for resource-limited areas. For public health prevention, schools can integrate anti-bullying efforts with social skill-building (to boost friendship quality) and rumination awareness curricula—creating a cost-effective, scalable system to reduce NSSI risk.

## 6 Limitations

Although this study clarifies the moderated mediation pathway between bullying victimization and NSSI, it has a key limitation related to omitted variables, which affects the reliability of the model’s path relationships. First, we only included rumination (a cognitive factor) and friendship quality (a peer factor) in the model, but ignored family-level variables (e.g., parenting styles, parental emotional support) and school-level variables (e.g., anti-bullying policies, teacher intervention)—factors that could alter the strength of core paths. For instance, positive parental support might ease post-bullying negative emotions, thereby weakening the link between bullying victimization and rumination; effective school anti-bullying policies could also reduce the direct impact of bullying on NSSI. The exclusion of these variables means the relationships we observed (e.g., the mediating role of rumination, the direct effect of bullying) may not fully reflect real-world dynamics, as they do not account for the buffering or exacerbating effects of family and school contexts. Second, the cross-sectional design prevents establishing causal relationships, further limiting the certainty of how these paths operate over time. Future studies integrating multi-level variables would help verify the stability of these paths and enhance the model’s validity. This study utilizes a cross-sectional research methodology. Although this method is able to reveal the relationship between variables at a particular point in time, providing the study with some immediate data and preliminary conclusions, its inherent limitation is that it is difficult to explore in depth the dynamic relationship between variables over time. This dynamic relationship is crucial to a comprehensive understanding of the nature of the research problem and the intrinsic connection between the variables. Therefore, future research can consider adopting a longitudinal tracking research method to conduct long-term follow-up observation and data collection on the same research subjects. In this way, the evolution of variables in different time stages can be explored more deeply, revealing potential causal relationships and other possible complex interactions, thus further verifying the reliability and stability of the results of the present study, providing more valuable insights and directions for subsequent related studies, and promoting the development of academic research to a deeper level.

This study was conducted from the perspective of adolescents’ emotional cognition, which helps to reveal adolescents’ cognitive-emotional mechanisms, but it failed to adequately consider the complex effects that external factors may have on the results of the study in the course of the research.

According to the Ecosystem Theory, individual development is essentially the result of the interaction between microsystems, mesosystems, and exosystems, but this study did not systematically examine the roles of family system variables such as family upbringing styles and levels of parental conflict, as well as the role of exosystem variables such as socio-cultural values, in the study results.

Meanwhile, the existing model only included peer friendship quality as a moderating variable, ignoring the potential impact of family relationships. As the primary site of adolescent socialization, elements such as family emotional support patterns and conflict resolution styles may influence individuals’ tendency to ruminate and expression of non-suicidal self-injurious behaviors through direct or indirect pathways, which were not included in the analysis. The failure to include them in the analysis resulted in limited explanatory power of the model.

In future research, we can incorporate microsystemic variables such as family upbringing style and parental conflict level, as well as external systemic variables such as socio-cultural values, to examine their moderating effects on the pathway of bullying and non-suicidal self-injurious behaviors. Secondly, by integrating Family Systems Theory and the Cognitive-Behavioral Model, we can analyze the dynamic mechanism through which family emotional support modes and conflict resolution modes influence individuals’ tendency to ruminate and then act on self-injurious behaviors using path analysis.

## 7 Conclusion

Being bullied not only has a direct effect on adolescents’ non-suicidal self-injurious behaviors, but can also act indirectly through ruminative thinking. Meanwhile, friendship quality can reduce the positive effect of rumination on adolescents’ non-suicidal self-injurious behaviors; that is, high-quality friendships can provide adolescents with adequate peer emotional support, so that adolescents will no longer be immersed in negative thinking, thus reducing the occurrence of non-suicidal self-injurious behaviors.

These findings deepen the understanding of the mechanism through which bullying affects non-suicidal self-injurious behaviors, and provide empirical evidence for the construction of a dynamic model of “bullying → rumination → non-suicidal self-injurious behaviors.” The results of this study have important implications for adolescent mental health interventions: by improving the quality of peer friendships and blocking the cognitive reinforcement process of rumination, the negative impact of bullying on non-suicidal self-injurious behaviors can be effectively weakened. It is suggested that improving the friendship support system and developing cognitive emotion regulation skills should be the focus of interventions to reduce the incidence of non-suicidal self-injurious behaviors in high-risk groups.

## Data Availability

The original contributions presented in the study are included in the article/supplementary material, further inquiries can be directed to the corresponding author.
